# Complement System Deficiencies in Elite Athletes

**DOI:** 10.1186/s40798-024-00681-0

**Published:** 2024-01-22

**Authors:** Libor Vítek, Jana Woronyczova, Veronika Hanzikova, Helena Posová

**Affiliations:** 1 Institute of Medical Biochemistry and Laboratory Diagnostics, 1st Faculty of Medicine, Charles University and General University Hospital in Prague, Kateřinská 32, 120 00 Prague, Czech Republic; 2 4th Department of Internal Medicine, 1st Faculty of Medicine, Charles University and General University Hospital in Prague, Prague, Czech Republic; 3 Sports Research Institute of the Czech Armed Forces, Prague, Czech Republic; 4 Blood Transfusion Unit, General University Hospital in Prague, Prague, Czech Republic

**Keywords:** Sport, Exercise, Elite athletes, Complement, Complementopathy

## Abstract

**Background:**

Although regular physical activity improves immune competency and reduces the prevalence of inflammatory diseases, strenuous training in elite athletes is associated with an increased susceptibility to infectious complications. Therefore, the objective of our study was to assess the routinely examined parameters of the complement system in elite athletes. The study was carried out in a cohort of elite athletes (*n *= 134) and healthy control subjects (*n *= 110). In all subjects, besides a routine laboratory check-up, serum concentrations of the C3 and C4 complement components, mannose-binding lectin (MBL), as well as activation of all three complement pathways were determined.

**Results:**

Compared to healthy controls, lower C3 and C4 complement component concentrations were observed in elite athletes (0.96 ± 0.1 vs. 1.08 ± 0.2 mg/L, and 0.18 ± 0.1 vs. 0.25 ± 0.1 mg/L, respectively, *p *< 0.05); with much higher frequency rates of C3 and C4 deficiencies in athletes (31.3 vs. 14.5%, and 6 vs. 0%, *p *< 0.05). Simultaneously, athletes had much higher frequency rates of deficiencies of activation of classical and alternative complement pathways; while, deficiency of activation of the lectin pathway was similar in both cohorts.

**Conclusions:**

We confirmed a high frequency of defects in the complement system in elite athletes. Lower concentrations of C3 and C4 complement components, with high frequencies of deficiencies of the classical and alternative complement activation pathways were the most prevalent disorder of the complement system in elite athletes. Further studies are needed to uncover the functional impacts of these observations upon the susceptibility to infectious diseases.

## Background

The role of regular physical training upon the immune system is a matter of debate. Although regular physical activity and frequent, short-lasting exercise enhance immune competency and reduces the prevalence of viral and bacterial infections as well as of chronic inflammatory disorders [1], the strenuous and long-lasting exhaustive training in elite athletes, particularly when with insufficient recovery, is associated with an increased susceptibility to infectious complications due to impairment of various stages of both the innate and adaptive immune systems [2–8]. Therefore, the relationship between increased exercise workload and susceptibility to upper respiratory tract infection has been modeled in the form of a ´J´-shaped curve suggesting that while engaging in moderate activity, immune functions may be enhanced, while excessive amounts of prolonged, high intensity exercise may impair immune function.

The complement system belongs to the older evolutionary parts of the immune system and consists of more than 50 proteins [9, 10]. The complement system which causes the lysis (bursting) of foreign and infected cells and also acts in chemotaxis-mediated phagocytosis, can be activated by three major pathways: classical (triggered by acute phase proteins), lectin (triggered, for example, by mannose-binding lectins (MBL)), and alternative (being robustly activated upon specific stimulation [11]). Complement also has an important role in the induction of antibody responses and serves as a bridge between the innate and adaptive immune systems [12]. Inherited complement deficiencies are quite rare in the general population, with a prevalence of about 1:1000 as reported in a large Japanese cohort [13], and further confirmed in other studies [14, 15]. Nevertheless, the states of low complement activity caused by a nonspecific modulation of a complement system, especially in the lectin activation pathway, is quite more frequent [13].

Several factors of the complement system are routinely examined in clinical practice, including the C3/C4 components, MBL, and/or activation of all three complement pathways. The C3 complement component participates in both classic and alternative pathways, and its deficiency can cause impairment of the immune response and a higher risk of infection [16]. While overstimulation of activity of the complement system can cause serious organ and tissue damage including organ rejection, asthma, multiple sclerosis, sepsis, or Alzheimer’s disease [17], its deficiency has been associated with increased susceptibility to infections [18] and autoimmune diseases [19].

Complement activation is also likely implicated in the pathogenesis of chronic fatigue syndrome and post-exercise malaise [20, 21], which is certainly of interest in the realm of elite sports.

Involvement of the complement system in post-effort immunity has also been documented [22]. Aerobic exercise can trigger various immune responses via activation of the alternative pathway [23]. As mentioned above, intensive physical activity can stimulate or suppress the immune response; the final outcome depends on many factors such as age and fitness level, triggering oxidative stress and the release of cortisol, catecholamines, insulin-like growth factor, and heat shock proteins [3, 24].

However, data published in the literature are quite heterogeneous. Therefore, the aim of our study was to assess those routinely examined parameters of the complement system in a cohort of elite athletes.

## Methods

### Subjects

The subjects involved in the study were recruited among healthy consecutive elite athletes (*n *= 134; M:F ratio = 1.4; mean age 23.2 ± 5 years, Table 1), and were regularly examined during preventive examination in the 4th Department of Internal Medicine, General University Hospital in Prague through 2017–2022. All athletes were members of either Czech national teams or centers of elite sport. During the examination, a complete laboratory baseline check-up was performed that included examination of the complement system, to search for susceptibility to infectious diseases. All of the examinations were carried out according to good standards of medical practice. Healthy blood donors from the Blood Transfusion Unit of the General University Hospital in Prague were used as a control group (*n *= 110; M:F ratio = 2.7; mean age 40 ± 11 years, Table 1). All subjects were of Caucasian origin.

**Table 1:** Parameters of complement system in elite athletes

Parameter	Athletes (*n *= 134)	Control population (*n *= 110)	*P* value
M/F (*n*)	77/57	80/30	
Age (years)	23.2 ± 5	40 ± 11	** < 0.05**
C3 (mg/dL, normal range: 0.9–1.8)	0.96 ± 0.1	1.08 ± 0.2	** < 0.001**
C3 deficiency (%)	31.3	14.5	** < 0.005**
C4 (mg/dL, normal range: 0.1–0.4)	0.18 ± 0.1	0.25 ± 0.1	** < 0.001**
C4 deficiency (%)	6.0	0	** < 0.05**
MBL (μg/L, normal range: 103–3308)	1141 ± 820	792 ± 590	** < 0.001**
Classical pathway activation (%, normal range: 66–100)	82.2 ± 15	93.9 ± 10	** < 0.001**
Classical pathway deficiency (%)	14.4	2.8	** < 0.05**
Alternative pathway activation (%, normal range: 25–100)	68.3 ± 20	78.1 ± 14	** < 0.001**
Alternative pathway deficiency (%)	4.8	0	** < 0.05**
Lectin pathway activation (%, normal range: 10–100)	44.3 ± 39	33.4 ± 34	NS
Lectin pathway deficiency (%)	27.9	34.9	NS

Data expressed as mean ± SD and compared using t test or Mann–Whitney Rank Sum test when non-normally distributed. Frequency rates of complement pathways deficiencies (determined as values below the normal ranges) were compared using Chi-square test

Significant difference are given in bold

*C3, C3* Complement component, *C4, C4* Complement component, *MBL* Mannose-binding lectin

Written informed consent was obtained from each human subject included in the study, and the study protocol was consistent with the ethical guidelines of the Declaration of Helsinki of 1975, as reflected in a priori approval by the Ethics Committee of the General University Hospital in Prague (No. 79/22).

### Laboratory Analyses

In all subjects, the standard serum biochemistry and complete blood count were determined by routine assays on automated analyzers (Cobas R8000 Modular analyzer, Roche Diagnostics GmbH, Mannheim, Germany; and Sysmex XN-550, Sysmex, Kobe, Japan, respectively).

Due to serious pre-analytical concerns related to individual complement component determinations [25, 26], blood serum samples were collected instantly after blood clotting and immediately analyzed to prevent complement activation in the whole blood [27]. The MBL level was measured using a commercially available ELISA kit (R&D Systems, MN, USA) according to the manufacturer’s instructions, with reference values between 103 and 3308 ng/mL. The MBL was examined in a subset of the athletes (consecutive athletes examined during 2019–2022, *n *= 93), and in all control subjects.

Nephelometric measurements to quantitatively determine serum concentrations of the C3 and C4 complement components were performed with a Nephelometer Siemens BN-II (Siemens Healthineers, Germany). The principle of this method is based on the light dispersion of circulating immune complexes that have passed through the serum sample. The intensity of the dispersed light is proportional to the C3 or C4 component concentration; quantification is made using specific standards of known concentrations. The reference values for C3 and C4 were 0.9–1.8 mg/L, and 0.1–0.4 mg/L, respectively.

For the complement pathway activity measurements blood serum samples were collected according to the manufacturer´s instructions of the analytic kits, *i.e.,* blood was allowed to clot in serum tubes for 60–65 min at room temperature (20–25 °C), blood samples were centrifuged and cell-free sera were transferred to clean tubes and immediately analyzed. The Wieslab^®^ Complement System Screen Kit (SVAR Life Science, Malmo, Sweden) was used for the qualitative determination of functional classical (66–100%), lectin (10–100%), and alternative (25–100%) complement pathways in human serum. The Wieslab Complement Classical Pathway assay combines the specific activation of the pathway with the use of labeled antibodies specific for a neoepitope of the terminal complement complex, C5b-9, produced as the result of complement activation; while, mannan and lipopolysaccharide are used for determination of activation of lectin and alternative complement pathways, respectively.

### Statistical Analyses

Data are expressed as mean ± SD and compared using *t*-test or Mann–Whitney Rank Sum test when nonnormally distributed. Frequency rates of complement pathways deficiencies were compared using Chi-square test. Relationships between some of the analyzed variables were evaluated by linear regression analyses; while, a Spearman correlation analysis was used to assess correlation between other selected variables. All analyses were performed with alpha set to 0.05. Statistics were calculated using SigmaPlot v. 14.5 (Systat Software Inc., San Jose, CA, USA).

## Results

The whole study was conducted on 134 healthy elite athletes, mainly from endurance and team sports (Table 2), and their data were compared to healthy control population.

**Table 2:** Athletes by sport discipline

Sports discipline	Sport	Number of subjects	Sex distribution (male/female)
Speed-endurance	Athletics (runs) Cycling Swimming Triathlon Pentathlon	24	12/12
Strength-endurance	Sprint canoeing Wild-water canoeing Rowing	28	13/15
Endurance	Biathlon Cross-country skiing	38	18/20
Speed-strength	Alpine skiing Athletics (throwing; jumping) Sport gymnastics Fencing	7	3/4
Games	Basketball Tennis Volleyball	34	30/4
Other	Shooting Dancing Wrestling	3	0/3

### Laboratory Analyses

The elite athletes examined during routine check-ups showed no differences in their physiological values of: serum mineralogram, blood proteins, inflammatory markers, kidney and liver function tests, thyroid hormones, or iron metabolites. Serum levels of IgG, IgM, and IgA were also within their normal physiological ranges in all athletes examined, similarly in the complete blood count with the exception of a mild neutropenia observed in 12.7% of the athletes (in the range of 1.43–2.0*10^9^/L). Neutrophil counts did not correlate either with serum concentrations of immunoglobulins, C3 and C4 complement components, or MBL (data not shown).

### Parameters of the Complement System

Compared to healthy controls, lower C3 and C4 complement component concentrations were observed in elite athletes (0.96 ± 0.1 vs. 1.08 ± 0.2 mg/L, and 0.18 ± 0.1 vs. 0.25 ± 0.1 mg/L, respectively, *p *< 0.001 for both comparisons, Table 1); with much higher frequency rates of C3 and C4 deficiencies in athletes (31.3 vs. 14.5%, and 6 vs. 0%, *p *< 0.005 and < 0.05, respectively, Table 1).

Simultaneously, athletes had much higher frequency rates of deficiencies of activation of classical and alternative complement pathways (82.2 ± 15 vs. 93.9 ± 10%, *p *< 0.001, Table 1); while, deficiency of activation of the lectin pathway was similar in both cohorts (44.3 ± 39 vs. 33.4 ± 34%, *p *> 0.05, Table 1).

**Fig. 1:**
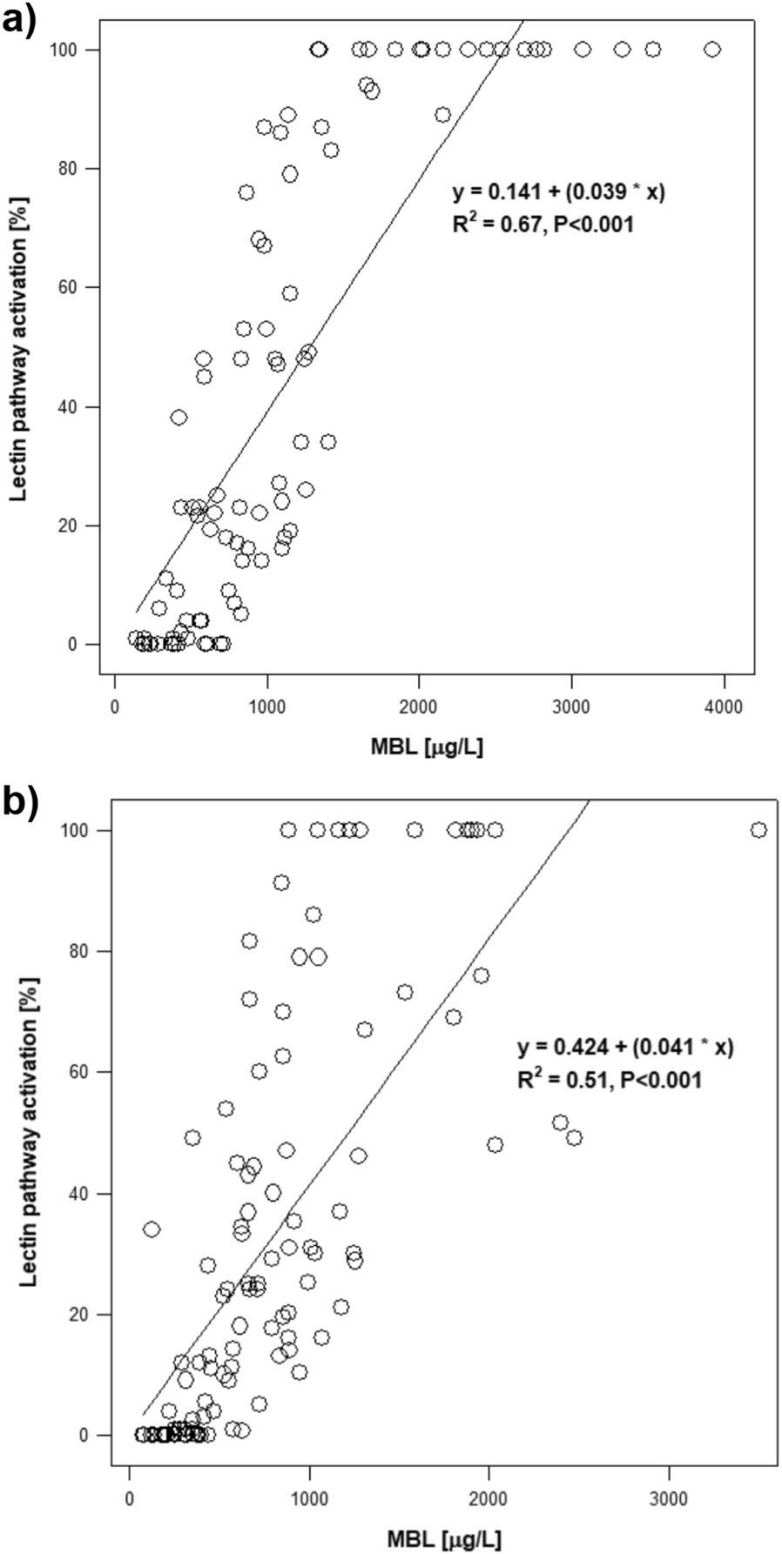
Relationship between activation of the lectin complement pathway and serum MBL concentrations in **a** athletes and **b** control subjects. MBL, mannose-binding lectin

Quite surprisingly, serum MBL concentrations were significantly higher in athletes compared to control subjects (1141 ± 820 vs. 792 ± 590 μg/L, *p *< 0.001, Table 1). A linear relationship between serum MBL concentrations and the degree of activation of the lectin pathway was present in both examined groups of subjects (Fig. 1).

A negative relationship between the serum concentrations of complement component C3 and C4 and IgM antibodies was observed (Table 3); while, parameters of the complement system were neither related to any parameter of the complete blood count, nor the sport discipline. The only exception was the lectin complement pathway, which was much less active in speed strength compared to speed endurance sports disciplines.

**Table 3:** Relationship between serum concentrations of C3 and C4 complement components and immunoglobulin levels

	IgG	IgM	IgA	IgE	IgG1	IgG2	IgG3	IgG4
C3	− 0.047	− 0.190	0.059	0.216	0.007	− 0.152	0.059	− 0.064
*P *value	> 0.05	**0.039**	> 0.05	**0.017**	> 0.05	> 0.05	> 0.05	> 0.05
C4	− 0.086	− 0.191	− 0.008	0.028	− 0.070	0.002	0.144	− 0.195
*P *value	> 0.05	**0.038**	> 0.05	> 0.05	> 0.05	> 0.05	> 0.05	**0.046**

Values represent Spearman correlation coefficient with respective *P* values

The pair of variables with positive correlation coefficients and *P* values below 0.05 tend to increase together. In the pairs with negative correlation coefficients and *P* values below 0.05, one variable tends to decrease; while, the other increases

Significant difference are given in bold

When the individual parameters of the complement system were compared between men and women, virtually no significant changes were observed between both sexes. Exactly the same observation was made for the neutrophil counts and immunoglobulin concentrations.

## Discussion

Despite there being some controversy, exhaustive and prolonged training in elite athletes is believed to be an increasing risk factor to infectious diseases [2–7] and generally recognized that even minor infections can result in a drop in exercise performance and the ability to sustain heavy training [28]. On the other hand, attempts thus far reported comparing resting immune function in athletes and non-athletes have failed to provide evidence that athletic training is linked to clinically important changes in immunity [29–32]; also given that any compelling robust epidemiological evidence is lacking, and with their only having been scarce studies evaluating the complement system [32].

Inherited complement deficiencies occurs rarely in the general population while states of low complement activity caused by a nonspecific modulation of a complement system, especially in the lectin activation pathway, is quite more frequent [13–15]. In fact, we have observed a high prevalence of lectin pathway activation deficiency in both control group as well as elite sport athletes (Table 1). Although MBL closely correlates with the complement system’s lectin activation pathway (as has both been demonstrated in healthy controls [18, 33] and immunodeficient patients [18]), no athlete or healthy subject in our current study was found to have low serum MBL concentrations (Table 1). Despite this observation, there still was a linear relationship between serum MBL concentrations and the degree of lectin pathway activation in both examined groups of subjects (Fig. 1).

However, in addition to this observation, a substantial number of elite athletes had a defect in other parts of their complement system including defects in the classical and alternative complement pathway activation as well as low serum concentrations of serum C3 and C4 concentrations.

It should be noted that in previous reports the low capacity for complement activation through the lectin pathway was significantly associated with an increase in the frequency of lower respiratory tract infections [18]. Interestingly, no defect in the classical and alterative complement pathway was observed in this study [18] consistent with a very low prevalence of deficiencies in these two activation pathways in our healthy subjects (prevalence of 2.8%, Table 1). However, deficiencies in these two complement pathways were much more common in elite athletes (prevalence of 19.2%, Table 1), which certainly can be a predisposing factor for infectious diseases.

In the studied athletes, we also found lower serum concentrations of the C3 and C4 components of the complement system. In a previous study, bouts of both aerobic and anaerobic exercises were associated with a rapid post-exercise drop in C3 and C4 complement components; however, which went back to pre-exercise levels very quickly [22]. In another study, the C3 and C4 resting state levels in long distance runners were significantly lower compared to sedentary controls; while, aerobic exercise led to increased C3 and C4 concentrations [34]. In another older report, the levels of activated C3 and C4 increased immediately after a 2.5 h running test; the complement most likely being activated as the result of a tissue debris clearance mechanism released during mechanical/metabolic tissue damage [35]. Complement activation with an increase in C3 and C4 complement component concentrations (having been within the normal range in the pre-exercise period) after strenuous running exercise has also been reported in another small Spanish study [36]. It is also likely that exercise-induced reductions in plasma volume might partially contribute to these post-exercise-induced increases in C3 and C4 complement component concentrations [37].

A very recent review paper summarizing 77 published studies covering more than 10,000 examined subjects revealed that the baseline C3 levels in athletes were mostly decreased due to long-term training, and quite surprisingly were associated with increased cardiorespiratory fitness [38], which is a finding consistent with our data.

Low levels of these complement components in elite athletes are likely to be due to accelerated consumption of immune complexes being increasingly formed during bouts of training [35, 39, 40], with complement proteins being consumed during the process of clearing circulating immune complexes. It is also known that low protein diets and low energy availability, often present in endurance athletes, are associated with decreased concentrations of C3 and C4 complement components [41, 42]; and the same is true for some micronutrient deficiencies, such as vitamin D [43].

In addition to impairment of the complement system, we have also observed mild asymptomatic neutropenia in relatively high number of our athletes (almost 13%), consistent with previously reported data [44]. It needs to be emphasized that both neutrophils and a complement system are inter-connected. In fact, neutrophils were demonstrated to activate both classical and alternative complement pathways which in turn activate neutrophils in a positive amplification loop [45, 46]. Hence, both relative neutropenia and impairment of complement system in elite athletes can contribute to their increased susceptibility to infectious complications.

Interestingly, supplementation of specific nutrients has been demonstrated to improve the complement system. In a study by Tang et al. [47] on healthy adults, an enriched mangosteen product containing multivitamins and essential minerals increased serum concentrations of C3 and C4 complement components.

It also should be noted, that the impaired complement system in elite athletes could be a risk factor for the development of not only infectious complications, but even of autoimmune diseases [19, 48], although there is so far no clinical evidence for this statement.

In addition, the complement system seems to have much deeper and wider consequences. In fact, recent discoveries revealed its intracellular roles in energy homeostasis (via modulation of glycolysis and mitochondrial functions) [10], or even in neuromuscular functions (via Wnt pathway) [49, 50]; leading to the coining of the name complosome for all these metabolic interactions, which are certainly important in elite sportspersons.

Our study has several limitations. First, the athletes were examined regardless the period/phase of training or competition, which could affect the data on complement system. Furthermore, determination of serum MBL concentrations was available not in the whole group of athletes, since the routine examination of this parameter started later. In addition, due to limited number of athletes in individual sport disciplines, the parameters of complement system could not be compared across different sports. Finally, individual susceptibility to infections has not been assessed in the current study. Future studies with the implementation of some of the questionnaire tools (such as the WURSS questionnaire [51]) would shed more light on the clinical relevance of complement deficiency in elite athletes.

## Conclusions

In summary, we have confirmed the high frequency of defects in the complement system in elite athletes. Lower concentrations of C3 and C4 complement components, with high frequencies of deficiencies of the classical and alternative complement activation pathways were the most prevalent disorder of the complement system in elite athletes. Further studies are needed to uncover the functional impacts of these observations upon the susceptibility to infectious diseases in elite athletes.

## Data Availability

Data will be made available on reasonable request.

## Abbreviation

MBL: Mannose-binding lectin
